# Study of Imaging Biomarkers as a Prognostic Factor and Guide in the Management of Diabetic Macular Oedema

**DOI:** 10.7759/cureus.73765

**Published:** 2024-11-15

**Authors:** Nikita Shrivastava, Vivek Som, Kavita Kumar

**Affiliations:** 1 Ophthalmology, Gandhi Medical College and Hamidia Hospital, Bhopal, IND

**Keywords:** diabetic macular oedema, imaging biomarkers, non-proliferative diabetic retinopathy (npdr), prognostic predictor, proliferative diabetic retinopathy (pdr)

## Abstract

*Background: *Diabetic macular oedema (DME) is a major cause of vision impairment in individuals with diabetes mellitus, characterised by fluid accumulation in the macula due to increased vascular permeability. The growing prevalence of diabetes worldwide has led to an increasing burden of DME on healthcare systems. While current treatment options such as anti-vascular endothelial growth factor (anti-VEGF) injections, corticosteroids, and laser therapy exist, the variability in patient responses highlights the need for reliable prognostic tools. Imaging biomarkers, particularly those identified using optical coherence tomography (OCT) and fluorescein angiography (FA), play a critical role in diagnosing and managing DME. This study evaluates the prognostic significance of these biomarkers in predicting disease progression and treatment outcomes.

*Materials and methods: *This prospective observational study was conducted at Gandhi Medical College and Hamidia Hospital, Bhopal, Madhya Pradesh, from August 2022 to June 2024. A total of 123 patients with Type II diabetes mellitus diagnosed with DME were included through consecutive sampling. Comprehensive assessments, including visual acuity, slit-lamp examination, fundus evaluation, FA, and OCT, were performed. Key imaging biomarkers, such as central subfield thickness (CST), disorganisation of retinal inner layers (DRIL), intraretinal cysts, hyperreflective foci, and vitreomacular interface (VMI) abnormalities, were evaluated. Correlations between biomarkers, best-corrected visual acuity (BCVA), metabolic markers (HbA1c, serum cholesterol), and disease severity were analysed using statistical tools, including the chi-square test and Pearson's correlation.

*Results: *The most common biomarkers observed were DRIL with external limiting membrane (ELM) disruption (38, 31%), intraretinal cysts with ELM disruption (36, 29.3%), and hyperreflective foci (28, 22.8%). VMI abnormalities were noted in 14 (11.4%) cases, while subretinal fluid with serous retinal detachment was present in seven patients (5.7%). Significant negative correlations were found between BCVA (LogMAR) and biomarkers, with intraretinal cysts (-0.526, p=0.003) and VMI abnormalities (-0.492, P=0.002) having the strongest associations. Higher glycosylated haemoglobin (HbA1c) levels were significantly associated with intraretinal cysts (P=0.014) and VMI abnormalities (P=0.042), while higher cholesterol levels correlated with hyperreflective foci (P=0.011) and subretinal fluid (P=0.014). Patients with proliferative diabetic retinopathy (PDR) exhibited worse visual outcomes and greater CST compared to those with non-proliferative diabetic retinopathy (NPDR).

*Conclusion: *Imaging biomarkers, particularly DRIL, intraretinal cysts, and VMI abnormalities, significantly correlate with visual acuity and metabolic control in DME patients. These findings underscore the importance of OCT in the prognostic assessment of DME and highlight the need for personalised treatment approaches based on biomarker profiles. Future studies should focus on long-term follow-up and explore the potential for integrating these biomarkers into clinical decision-making to improve patient outcomes.

## Introduction

Diabetic macular oedema (DME) is a common complication of diabetic retinopathy and one of the leading causes of vision impairment in individuals with diabetes mellitus [1]. It is characterised by the accumulation of fluid in the macula due to increased vascular permeability, resulting in retinal thickening and visual loss [2]. With the growing prevalence of diabetes worldwide, the burden of DME on healthcare systems and affected individuals continues to rise [3]. Effective management of DME is critical in preventing vision loss and improving the quality of life for patients [4,5]. Current treatment options include anti-vascular endothelial growth factor (anti-VEGF) injections, corticosteroids, and laser therapy, but responses can vary, highlighting the need for reliable prognostic tools to personalise treatment approaches [4,6].

Imaging biomarkers have become valuable tools in the diagnosis and management of retinal diseases, including DME [7]. Optical coherence tomography (OCT) and fluorescein angiography (FA) are frequently used imaging modalities that provide detailed insights into retinal structure and vascular changes [8]. OCT, in particular, allows for high-resolution cross-sectional imaging of the retina, enabling precise measurements of retinal thickness and the identification of morphological features associated with DME. While FA provides information on retinal perfusion and leakage, it is an invasive procedure and is gradually being replaced by OCT and optical coherence tomography angiography (OCT-A) for retinal vascular pathologies [7,8].

Recent advancements in imaging technology have led to the identification of several potential biomarkers for DME, such as central subfield thickness (CST), intraretinal cysts, subretinal fluid, hyperreflective foci, disorganisation of the retinal inner layers (DRIL), and vitreomacular interface (VMI) abnormalities. Understanding the prognostic significance of these biomarkers can help predict disease progression, treatment response, and visual outcomes [8,9]. This study aims to evaluate the role of imaging biomarkers in predicting prognosis and guiding the management of DME. By analysing a cohort of patients using OCT, we seek to identify key biomarkers that correlate with disease severity and treatment outcomes, contributing to the development of personalised treatment strategies for improved patient care and outcomes.

## Materials and methods

This prospective observational study was conducted in the Department of Ophthalmology at Gandhi Medical College and Hamidia Hospital, Bhopal, Madhya Pradesh, from August 2022 to June 2024, focusing on patients with type II diabetes mellitus screened for diabetic macular oedema (DME). Screening was conducted in two stages: initial screening was performed by a resident doctor, followed by confirmation by a consultant (professor) before patients were officially included in the study. All diagnostic parameters and measurements were also confirmed by the consultant to ensure accuracy. Patients either presented directly to the Outpatient Department (OPD) or were referred from other departments within the hospital. Inclusion criteria included patients diagnosed with DME who provided written informed consent and had sufficient OCT image quality for analysis. Forty patients with dense cataracts or vitreous haemorrhage, which impaired OCT quality, were excluded from the study. Additionally, 17 patients declined to participate, resulting in a final sample size of 123 patients enrolled through consecutive sampling.

Prior to study initiation, ethical clearance was granted by both the Institutional Review Board (IRB) and the Institutional Ethics Committee (IEC) of Gandhi Medical College and Hamidia Hospital, Bhopal, under reference number 32243/MC/IEC/2022, dated 13 August 2022. Informed written consent was obtained from all participants (see appendices), and strict privacy and confidentiality protocols were enforced to protect participants’ personal and medical information.

Each patient underwent a comprehensive screening process, starting with a visual acuity assessment, followed by slit lamp and fundus examinations. After informed consent was secured, fundus fluorescein angiography (FFA) was performed to evaluate retinal capillary leakage patterns, foveal avascular zones, microaneurysms, and neovascularisation. Optical coherence tomography (OCT) was then used to assess retinal structural changes, including retinal thickening, disorganisation of retinal inner layers (DRIL), intraretinal cysts, subretinal fluid, and serous retinal detachment. The eye with the worse best-corrected visual acuity (BCVA) was selected for analysis, ensuring that only the most affected eye contributed to the evaluation. This method provided a comprehensive understanding of abnormalities in eyes with significant vision impairment.

To investigate metabolic influences, metabolic markers such as glycosylated haemoglobin (HbA1c) and serum cholesterol levels were assessed to explore their association with imaging biomarkers and BCVA. For BCVA analysis, measurements were converted to LogMAR values (Logarithmic Minimum Angle of Resolution) [10], a scale that quantifies visual acuity based on letters missed rather than letters correctly identified. A perfect LogMAR score (equivalent to 6/6 vision) is 0.0, while each missed letter increases the score by 0.02, indicating reduced acuity. For instance, if a patient only reads the 6/7.5 line, missing five letters on the 6/6 line, their LogMAR score would be 0.1. Higher LogMAR values correspond to worse vision (e.g., 6/9 is 0.2), while scores below zero (e.g., 6/4.8 is -0.1) indicate better-than-standard acuity. A complete table of visual acuity notations can be found in the appendices (Table 8). Retinal abnormalities such as DRIL, cysts, hyperreflective foci, and subretinal fluid were systematically counted for each patient, providing a quantitative assessment of abnormalities per eye.

Following diagnostic procedures, treatment was administered per the Early Treatment Diabetic Retinopathy Study (ETDRS) [11] guidelines, a standard protocol for managing DME. The study’s primary objective was to examine relationships between imaging biomarkers (from FFA and OCT), diabetes duration and severity, metabolic control (HbA1c and cholesterol), and BCVA in DME patients. By analysing these variables, the study aimed to clarify the effects of prolonged hyperglycaemia and dyslipidemia on DME progression and visual outcomes.

For statistical analysis, Jamovi Solid Version 2.3.28 was employed. Quantitative variables were expressed as means and standard deviations, while categorical data were presented as numbers and percentages. The chi-square or Fisher's exact test was used to analyse categorical data, and means were compared using the t-test. Prior to mean comparisons, Q-Q plots assessed normality, and Levene's test checked for homogeneity. Correlation analysis was also conducted, with Q-Q plots used to confirm normality and scatter plots to validate the linearity assumption. A p-value of less than 0.05 was considered statistically significant, supporting the robustness of the study’s conclusions. The correlation coefficients were interpreted as follows: strong positive (1 > r ≥ 0.8), moderate positive (0.8 > r ≥ 0.4), and weak positive (0.4 > r > 0). Negative correlations were classified as weak (0 > r ≥ -0.3), moderate (-0.3 > r ≥ -0.6), strong (-0.8 > r ≥ -1), with -1 indicating perfect negative correlation.

## Results

The study included a total of 123 patients, with the following distribution by age group: the majority of patients were more than 60 years old (56, 45.5%), followed by those aged 51-60 years (48, 39%), and a small proportion of patients were 40 years or younger (3, 2.5%). The gender distribution was nearly equal, with males (64, 52%) and females (59, 48%). The mean duration of diabetes among the patients was 11.08 ± 4.26 years.

In terms of biomarkers, the most commonly observed were disorganisation of the retinal inner layers with external limiting membrane (ELM) disruption (38, 31%) and intraretinal cysts with ELM disruption (36, 29.3%). Additional biomarkers included hyperreflective foci (28, 22.8%), vitreomacular interface (VMI) abnormalities (14, 11.4%), and subretinal fluid with serous retinal detachment (7, 5.7%) (Table 1).

**Table 1:** Frequency distribution of biomarkers (N=123)

Biomarker	Frequency (%)
Disorganisation of retinal inner layers with external limiting membrane (ELM) disruption	38 (31)
Intraretinal cysts with ELM disruption	36 (29.3)
Hyperreflective foci	28 (22.8)
Vitreo-macular interface (VMI) abnormalities	14 (11.4)
Subretinal fluid with serous retinal detachment	7 (5.7)

Table 2 highlights the distribution of various biomarkers across visual acuity categories in patients with diabetic macular oedema (DME). The chi-square test indicates a statistically significant association between the presence of biomarkers and visual acuity categories.

**Table 2:** Biomarkers across the visual acuity categories (N=123) ELM=External limiting membrane; VMI=vitreo-macular interface; RD=retinal detachment χ^2^=35.4; degrees of freedom (df)=8; P-value: < 0.001

Biomarker	6/12 - 6/24	6/60 - 6/24	< 6/60
Disorganisation of retinal inner layers with ELM disruption	1	10	23
Intraretinal cysts with ELM disruption	10	13	9
Hyperreflective foci	7	19	2
VMI abnormalities	0	3	4
Subretinal fluid with serous RD	0	6	8

The results from Table 2 align with the findings in Table 3. In the Materials and Methods section, we detailed the conversion of best-corrected visual acuity (BCVA) to LogMAR and the process of quantifying retinal abnormalities to transform qualitative observations into quantitative data. This provided two quantitative variables for comparison: visual acuity and the count of biomarkers in the eye with the worse BCVA.

Table 3 details the correlations between specific retinal biomarkers and best-corrected visual acuity (BCVA) on the LogMAR scale in patients with diabetic macular oedema (DME), including 95% confidence intervals (CIs) to indicate the precision of these estimates. All variables were normally distributed, and linearity assumptions were confirmed using scatter plots. Disorganisation of retinal inner layers, particularly with ELM disruption, shows a mild but statistically significant negative correlation with BCVA (Pearson coefficient -0.192; 95% CI: -0.357 to -0.015), suggesting that increased retinal disorganisation is associated with poorer visual outcomes. Intraretinal cysts display a stronger correlation (-0.526; 95% CI: -0.643 to -0.385), indicating a substantial impact on visual acuity as the presence of these cysts increases. Other significant findings include hyperreflective foci (-0.277; 95% CI: -0.433 to -0.105) and abnormalities in the vitreoretinal interface (VMI) (-0.492; 95% CI: -0.615 to -0.345), both associated with decreased vision. Subretinal fluid with serous retinal detachment shows a non-significant trend towards poorer vision (-0.666; 95% CI: -0.754 to -0.554, P = 0.088), indicating a possible association that did not reach statistical significance in this sample. Finally, central retinal thickness over 400 microns approaches significance (-0.468; 95% CI: -0.596 to -0.317, P = 0.051), suggesting it may contribute to reduced vision. Overall, these findings emphasise the varying degrees of influence these structural biomarkers have on visual outcomes in DME patients, with certain biomarkers contributing more to vision loss.

**Table 3:** Correlation between biomarkers and best-corrected visual acuity (BCVA) (LogMAR) CI=Confidence interval; ELM=external limiting membrane; VMI=vitreo-macular interface; RD=retinal detachment

Biomarker	BCVA (LogMar)	Pearson Coefficient	95% CI	P-value
Disorganisation of retinal inner layers with ELM disruption	1.30	-0.192	-0.357 to -0.015	0.001
Intraretinal cysts with ELM disruption	1.0	-0.526	-0.643 to -0.385	0.003
Hyperreflective foci	0.9	-0.277	-0.433 to -0.105	0.009
VMI abnormalities	1.47	-0.492	-0.615 to -0.345	0.002
Subretinal fluid with serous RD	1.30	-0.666	-0.754 to -0.554	0.088
Central subfield thickness (>400 microns)	1.17	-0.468	-0.596 to -0.317	0.051

Table 4 presents the distribution of cases based on the duration of diabetes. The mean duration of the disease among patients was 11.08±4.26 years, with the majority (62, 50.4%) having a disease duration of 10 years or more. Biomarkers like VMI abnormalities and DRIL were more frequently observed in patients with a longer duration of diabetes.

**Table 4:** Duration of diabetes and biomarkers (N=123) DRIL=Disorganisation of retinal inner layers; ELM=external limiting membrane; VMI=vitreo-macular interface; RD=retinal detachment

Duration of diabetes	Number of cases (%)	Biomarkers
<5 years	27 (22)	Hyperreflective foci and intraretinal cysts
5-10 years	34 (27.6)	Intraretinal cysts, hyperreflective foci, and DRIL
>10 years	62 (50.4)	VMI abnormalities, intraretinal cysts, DRIL, and ELM disruption

Patients with DME and proliferative diabetic retinopathy (PDR) had significantly higher central subfield thickness (CST) (521 vs. 459, P = 0.007) and worse best-corrected visual acuity (BCVA) (0.80 vs. 0.59, P < 0.001) compared to those with non-proliferative diabetic retinopathy (NPDR). Additionally, VMI abnormalities and larger cyst sizes (>100 microns) were more frequently observed in the PDR group (P < 0.01 and P = 0.01, respectively). Other parameters, such as subretinal fluid and hyperreflective foci, did not show significant differences between the groups (Table 5).

**Table 5:** Parameters across different stages of diabetic retinopathy (N=123) CST=Central subfield thickness; BCVA=best corrected visual acuity; DME=diabetic macular oedema; NPDR=non-proliferative diabetic retinopathy; PDR=proliferative diabetic retinopathy; ELM=external limiting membrane; VMI=vitero-macular interface; RD=retinal detachment

Parameter	DME with NPDR (n=52)	DME with PDR (n=71)	P-value
CST (mean ± SD)	459	521	0.007
BCVA	0.59	0.80	< 0.001
ELM disruption	3	5	0.03
Intraretinal cystic spaces	14	18	0.05
Hyperreflective foci	15	13	0.12
VMI abnormalities	1	10	< 0.01
Subretinal fluid	8	6	0.09
Size of cysts >100 microns	8	16	0.01

Higher glycosylated haemoglobin (HbA1c) levels were significantly associated with intraretinal cystic spaces (P = 0.014) and VMI abnormalities (P = 0.042). Other biomarkers did not show significant correlations with glycosylated haemoglobin (HbA1c) levels, as shown in Table 6.

**Table 6:** Association between biomarkers and HbA1c levels (N=123) HbA1c=Glycosylated haemoglobin; SD=standard deviation; RD=retinal detachment; VMI=vitreo-macular interface

Biomarker	HbA1c (%) (Mean ± SD)	P-value
Disorganisation of retinal layers	8.14 ± 0.65	0.071
Intraretinal cystic spaces	8.56 ± 0.77	0.014
Hyper reflective foci	7.98 ± 0.67	0.308
Subretinal fluid with serous RD	7.96 ± 0.84	0.767
VMI abnormalities	8.91 ± 0.81	0.042

Higher cholesterol levels were significantly associated with hyperreflective foci (P = 0.011) and subretinal fluid with serous retinal detachment (P = 0.014), as shown in Table 7. Other biomarkers, such as disorganisation of retinal layers and intraretinal cystic spaces, did not show significant correlations with cholesterol levels.

**Table 7:** Association between biomarkers and cholesterol levels (N=123) RD=Retinal detachment; VMI=vitreo-macular interface

Biomarker	Cholesterol (mg/dL) (Mean ± SD)	P-value
Disorganisation of retinal layers	238±45	0.098
Intraretinal cystic spaces	250±48.2	0.442
Hyper reflective foci	288.4±33	0.011
Subretinal fluid with serous RD	279.1±48	0.014
VMI abnormalities	284.7±46.45	0.308

## Discussion

The 2007 Diabetic Retinopathy Clinical Research Network publication [12] highlighted the wide range of visual acuity (VA) observed for a given degree of macular oedema, noting only a modest correlation between OCT-measured central retinal thickness (CRT) and VA. This suggests that factors beyond central retinal thickness contribute to visual deterioration in the setting of macular oedema. Speculations on these contributing factors include photoreceptor dysfunction, disrupted retinal structures, and accumulated hard exudates. Our findings align with the hypothesis that visual acuity outcomes in diabetic macular oedema are multifactorial, influenced by demographic factors and novel imaging biomarkers.

In our study, the mean age of patients was 63.4 years, consistent with other studies, such as Borrelli et al. (2023) [13], which reported a mean age of 61.4 years among DME patients undergoing anti-VEGF therapy. Similarly, Zur et al. (2018) [14] identified a significant prevalence of DME in older adults (mean age 64.8 years). The higher susceptibility in older populations could be due to prolonged exposure to hyperglycemia, leading to microvascular damage and subsequent retinopathy changes. This emphasises the importance of early detection and management of diabetes to mitigate the risk of developing DME in later years.

Our study observed a slight male preponderance among diabetic subjects, with 64 (52%) males and 59 (48%) females. This aligns with findings from Raman et al. (2010) [15], who also reported a male predominance in their studies.

The mean duration of diabetes mellitus in our study was 11.08±4.26 years, with most patients (62, 50.4%) having a disease duration of 10 years or more. This finding is corroborated by other studies, such as those by Wang et al. (2022) [16], which found that younger age at diagnosis of diabetes is an independent risk factor for diabetic retinopathy due to longer exposure to increased glycosylated haemoglobin levels. Berrabeh et al. (2023) [17] reported mean durations of diabetes mellitus of 7.23 ± 5.68 years in no DR, 9.83 ± 5.43 years in NPDR, and 10.89±4.29 years in PDR groups, indicating that disease severity increases with duration.

In our study, patients with specific biomarkers exhibited different HbA1c levels. Those with intraretinal cystic spaces had a mean HbA1c of 8.56% (p = 0.014), while those with VMI abnormalities had a mean HbA1c of 8.91% (p = 0.042). Studies by Borrelli et al. (2023) [13], Munk et al. (2022) [18], and Kim et al. (2019) [19] have also noted the relationship between HbA1c levels and OCT biomarkers, supporting that poor glycaemic control contributes to structural abnormalities.

Our study found that certain biomarkers correlated with higher mean cholesterol levels. Hyperreflective foci were associated with a mean cholesterol level of 288.36 mg/dL (p = 0.011). This is consistent with Kim et al. (2019) [19] and Arthi et al. (2021) [20], who demonstrated correlations between hyperreflective foci, hard exudates, and deranged lipid profiles, indicating potential benefits from lipid-lowering drugs.

The most common biomarker in our study was disorganisation of retinal inner layers (DRIL) with disrupted ELM (38, 31%), followed by intraretinal cystic spaces (36, 29.3%) and hyperreflective foci (28, 22.8%). Subretinal fluid was the least common (7, 5.7%). These findings align with studies by Costanzo et al. 2023) [21], Raman et al. (2010) [15], and Huang et al. (2021) [22], which reported similar observations.

We found significant negative correlations between BCVA and specific biomarker combinations, such as intraretinal cysts with DRIL and disrupted ELM or VMI abnormalities with intraretinal cysts/hyperreflective foci/DRIL. Our findings are supported by studies by Li et al. (2020) [23] and Das et al. (2018) [24] who also noted that these biomarkers are associated with decreased visual acuity.

In our study, patients with proliferative diabetic retinopathy (PDR) had higher CST (521 ± 101) and worse BCVA (0.80±0.11) compared to those with non-proliferative diabetic retinopathy (NPDR) (CST of 459±95 and BCVA of 0.59±0.09). This is consistent with studies such as Mehta et al. (2018) [25], which emphasise the role of increased CST in more severe forms of DME and its correlation with disease progression.

The strengths of this study include its detailed analysis of imaging biomarkers, offering valuable insights into their prognostic value in diabetic macular edema. The use of advanced imaging techniques, specifically OCT, provides precise retinal assessments, while the comprehensive data collection - covering demographics, glycemic control, and cholesterol levels - enhances the study's overall rigour. However, the study is limited by its relatively small sample size, single-center design, lack of longitudinal follow-up, and reliance on a single imaging modality. Many potential confounding factors like treatment variations, presence of other ocular comorbidities etc. were not considered and adjusted for.

## Conclusions

This study demonstrates the complex interplay of factors influencing vision loss in diabetic macular oedema (DME), highlighting that visual outcomes are affected not only by central retinal thickness but also by specific retinal biomarkers, patient demographics, and metabolic indicators. Key biomarkers identified - such as disorganisation of retinal inner layers (DRIL), disrupted external limiting membrane (ELM), and intraretinal cysts - were significantly linked to poorer visual acuity. Elevated HbA1c and cholesterol levels were also associated with structural retinal abnormalities, underscoring the potential benefit of strict glycaemic and cholesterol level management in reducing retinal damage among DME patients. Our findings align with other research, showing that older age, prolonged diabetes duration, and male gender are linked with greater DME severity.

This study reinforces the predictive value of imaging biomarkers, particularly through OCT, as essential tools for assessing disease severity and guiding treatment in DME. While this research provides valuable insights, future studies should consider addressing confounding factors such as treatment variations, coexisting ocular conditions, and systemic comorbidities, which may influence outcomes. Additionally, the limitations of a single-centre design and lack of longitudinal follow-up highlight the need for larger, multi-centre studies with extended monitoring. By accounting for these factors, future research can further validate these biomarkers' prognostic value, contributing to more personalised and effective treatment strategies for DME patients.

## Appendices

**Table 8:** BCVA-LogMAR conversion scale

Metre	Decimal	LogMAR
6/60	0.10	1.00
6/48	0.125	0.90
6/38	0.16	0.80
6/30	0.20	0.70
6/24	0.25	0.60
6/19	0.32	0.50
6/15	0.40	0.40
6/12	0.50	0.30
6/9.5	0.63	0.20
6/7.5	0.80	0.10
6/6	1.00	0.00
6/4.8	1.25	-0.10
6/3.8	1.60	-0.20
6/3	2.00	-0.30

Patient information sheet

*Title:* Study of imaging biomarkers as a prognostic factor and guide in the management of diabetic macular oedema

*Principal investigator:* Dr Nikita Shrivastava

*Location:* Bhopal

Date:

*Introduction:* You are invited to take part in the study of imaging biomarkers as a prognostic factor and guide in the management of diabetic macular oedema. Before you decide, if you want to participate, we would like you to understand why the study is being done, why your participation is needed, and how your information will be used.

What is the purpose of this research?

Diabetes mellitus is one of the oldest diseases which affects vision and can even progress to blindness, if diagnosed at a later stage. Our purpose is to diagnose diabetic macular oedema using various imaging modalities like FFA and OCT so that we can catch the disease and start appropriate treatment for the same.

Do I have to take part in the research?

It is up to you to decide whether or not to be a part in this study. If you do decide to take part, you will be given all relevant information regarding this study. And if you agree to participate and sign the consent form, you will be made a part of this study.

If you change your mind later, you can withdraw from this study at any stage, for any reason.

What do I have to do?

You have to come to our hospital for examination on given date and time, which can be more than two or three times.

What are the possible benefits and risks of taking part?

The benefit of taking part is that your eye will be thoroughly examined and risk factors and progression of disease will be noted. There is no risk of taking part in this study.

What do I do if I wish to withdraw from this research?

You will be given a withdrawal form to sign and you can easily withdraw to participate further in the research project and understand that such withdrawal will not jeopardize your future health care.

How is the study being conducted?

The study is being conducted in Gandhi Medical College on outpatient basis. Patient of diabetic macular edema of age 40 to 75 years will be selected and various tests will be done, which include visual acuity, FFA and OCT and other routine tests.

What will happen to the information you gather?

The information gathered will be studied for research purposes.

Will there be any financial assistance given for taking part in the study?

There will not be any financial assistance provided to participate in the research.

Further information whom to contact?

Name           :         Dr. Nikita Shrivastava

Address        :         17-A, Neelam Colony, Behind Lily D Mart Jahangirabad, Bhopal M.P.

Mobile No.   :         xx99xxxxxx

Email           :         nikitashrivastava41@gmail.com

Informed Consent form to participate in clinical trial/research (main study)

*Study Title:* Study of imaging biomarkers as a prognostic marker and guide in the management of diabetic macular edema

Study Number:

Participant’s Initials:

Participant’s Name:                                                         

Date of Birth/Age:                                               

1. I understand that I am being invited to take part in the research study. I confirm that I have read/ been read to and understood the information sheet dated for the above study and have had the opportunity to ask questions.

2. I understand that my participation in the study is voluntary and that I am free to withdraw at any time, without giving any reason, without my medical care or legal rights being affected.

3. I understand the risks and potential benefits of this research study that were explained to me. I freely give my consent to take part in the research study described in this form.

4. I understand that the Sponsor of the research study, others working on the Sponsor’s behalf, IEC and the regulatory authorities will not need my permission to look at my health records both in respect of the current study and any further research that may be conducted in relation to it, even if I withdraw from the trial. I agree to this access. However, I understand that my identity will not be revealed in any information released to third parties or published.

5. I agree not to restrict the use of any data or results that arise from this study provided such a use is only for scientific purpose(s).

6. I agree to take part in the above study.

I have read/have been read the above information and agreed to participate in this study. I have received a copy of this form.

Participant’s name:

Participant’s signature/thumb:

Impression and date:

Address:

Qualification:

Phone no:
